# Complement-Dependent Mechanisms and Interventions in Periodontal Disease

**DOI:** 10.3389/fimmu.2019.00406

**Published:** 2019-03-12

**Authors:** George Hajishengallis, Tetsuhiro Kajikawa, Evlambia Hajishengallis, Tomoki Maekawa, Edimara S. Reis, Dimitrios C. Mastellos, Despina Yancopoulou, Hatice Hasturk, John D. Lambris

**Affiliations:** ^1^Department of Microbiology, Penn Dental Medicine, University of Pennsylvania, Philadelphia, PA, United States; ^2^Division of Pediatric Dentistry, Department of Preventive and Restorative Sciences, Penn Dental Medicine, University of Pennsylvania, Philadelphia, PA, United States; ^3^Research Center for Advanced Oral Science, Graduate School of Medical and Dental Sciences, Niigata University, Niigata, Japan; ^4^Department of Pathology and Laboratory Medicine, Perelman School of Medicine, University of Pennsylvania, Philadelphia, PA, United States; ^5^Division of Biodiagnostic Sciences and Technologies, National Center for Scientific Research “Demokritos”, Athens, Greece; ^6^Amyndas Pharmaceuticals, Glyfada, Greece; ^7^Center for Clinical and Translational Research, Forsyth Institute, Cambridge, MA, United States

**Keywords:** complement, C3, therapeutics, compstatin Cp40, AMY-101, primate models, inflammation, periodontitis

## Abstract

Periodontitis is a prevalent inflammatory disease that leads to the destruction of the tooth-supporting tissues. Current therapies are not effective for all patients and this oral disease continues to be a significant public health and economic burden. Central to periodontal disease pathogenesis is a reciprocally reinforced interplay between microbial dysbiosis and destructive inflammation, suggesting the potential relevance of host-modulation therapies. This review summarizes and discusses clinical observations and pre-clinical intervention studies that collectively suggest that complement is hyperactivated in periodontitis and that its inhibition provides a therapeutic benefit. Specifically, interception of the complement cascade at its central component, C3, using a locally administered small peptidic compound (Cp40/AMY-101) protected non-human primates from induced or naturally occurring periodontitis. These studies indicate that C3-targeted intervention merits investigation as an adjunctive treatment of periodontal disease in humans.

## Introduction

**Figure 1:**
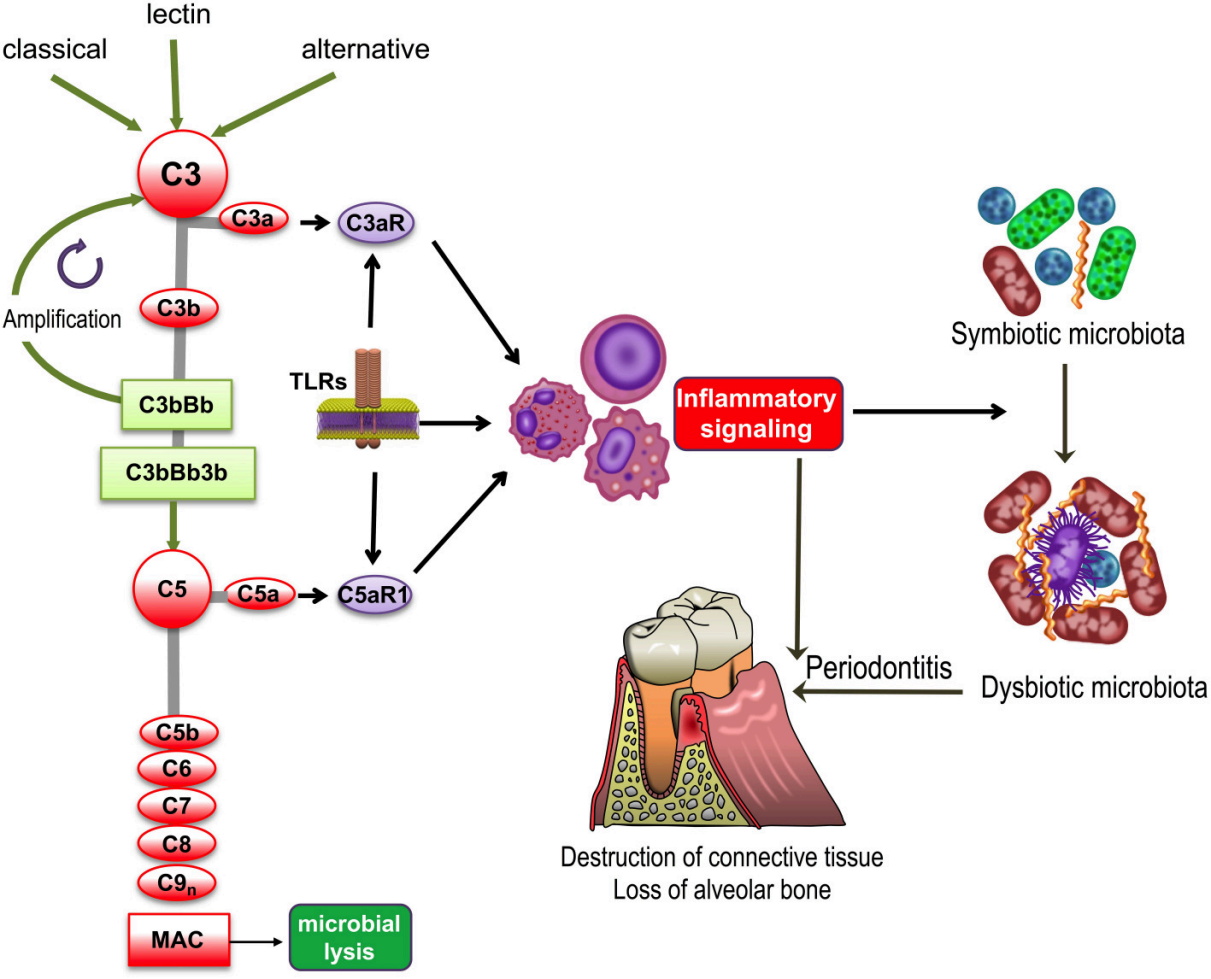
Complement and TLR involvement in dysbiosis and inflammatory bone loss in periodontitis. The classic, lectin, and alternative pathways converge at and activate the central complement component, C3, resulting in the generation of various effectors, such as, the C3b opsonin, the inflammatory anaphylatoxins C3a and C5a, and the C5b-9 membrane attack complex (MAC). The C3a and C5a activation fragments activate respectively C3aR and C5aR1, which cross-talk with Toll-like receptors (TLRs) and synergistically activate inflammatory leukocytes. TLRs also upregulate the expression of C3aR and C5aR1. Inflammation induced by complement-TLR crosstalk interactions not only causes gingival tissue destruction and bone loss in periodontitis but also contributes to the remodeling of a symbiotic microbiota into a dysbiotic one, thereby further potentiating destructive inflammation.

Complement represents an interactive network of soluble, cell surface-associated and intracellular molecules that activate, amplify, and regulate immunity and inflammation (1, 2). In addition to the classic serum proteins (C1-9), the network contains overall some 50 proteins, including pattern-recognition molecules, convertases and other proteases, receptors and regulators. Complement activation is initiated via distinct pathways, the classical, lectin, or alternative (Figure 1). The classical and lectin pathways are activated following the binding of complement-associated pattern-recognition molecules (e.g., C1q and mannose-binding lectin, respectively) to immune complexes (classical pathway) or to carbohydrate moieties exposed on microbial or damaged/necrotic host cells (lectin pathway). The alternative pathway is initiated by a tick-over mechanism and moreover amplifies the initial response induced by the other two complement pathways.

All three pathways converge at the central component of the complement system, C3, the activation of which leads to the generation of effectors that facilitate the ability of antibodies and phagocytes to clear microbial pathogens (via C3b opsonization), induce chemotaxis and inflammation (via the C3a and C5a anaphylatoxins), and lyse susceptible microbial targets (via the C5b-9 membrane attack complex [MAC]) (Figure 1). Furthermore, in cooperation with other immune and physiological systems, complement contributes to normal tissue and organ development, integrates and coordinates innate and adaptive immunity, mediates apoptotic cell clearance, and promotes tissue repair following injury (1, 3). In recent years, it has also become increasingly appreciated that complement is not exclusively produced in the liver and its actions are not restricted in the intravascular and extracellular compartments. Indeed, it has been shown that complement components can be produced locally by resident tissue cells as well as by recruited leukocytes. Moreover, an intracellular complement system was identified and shown to have novel homeostatic and immune functions, such as regulation of CD4^+^ T cell activation (2, 4).

Despite its importance in homeostatic immunity, complement may become dysregulated or excessively activated (e.g., due to host genetic or microbial virulence factors), thereby turning from a homeostatic to a pathological effector that can drive or exacerbate a number of disorders, such as cancer or inflammatory diseases, including periodontitis (Figure 1) as discussed in detail in the next section (5–8). Periodontitis is a common chronic inflammatory disease that causes destruction of the periodontium, i.e., the tissues that surround and support the teeth, namely, gingiva, periodontal ligament, and alveolar bone. If left untreated, periodontitis can lead to tooth loss and possibly impaired mastication (9). It is estimated that severe periodontitis afflicts ~10% of adults (10, 11). In its severe form, periodontal disease is associated with elevated risk for certain systemic conditions, such as atherosclerosis (12).

Recent evidence from clinical microbiome studies and mechanistic studies in animal models have shown that periodontitis is a dysbiotic disease rather than an infection attributed to a select few species (13–15). Connective tissue damage and loss of alveolar bone is mediated by a dysregulated and excessive inflammatory response, which includes components of both innate and adaptive immunity but fails to control the dysbiotic microbial challenge that induced it (16). In fact, the destructive periodontal inflammatory response is exploited by the dysbiotic microbial communities to procure nutrients from tissue breakdown products (17, 18). The fact that the disease is predominantly mediated by the host inflammatory response and that inflammation is necessary to support dysbiotic microbial communities justifies the rationale for developing host-modulation strategies to treat periodontitis. Such novel interventions may be used as adjuncts to improve current therapies (e.g., mechanical removal of the pathogenic biofilm), which are not always sufficient to treat periodontitis (19–22), thus accentuating its significant public health and economic burden (9, 11, 23, 24). Here, we discuss clinical and preclinical studies that have collectively linked complement overactivation to periodontitis and provided a mechanistic understanding of this relationship, paving the way to complement-targeted therapies to treat this oral inflammatory disease with strong associations to increased risk for other systemic conditions.

## Clinical Studies

The possible involvement of complement in human periodontitis was first recognized in the 1970s and 1980s by histological and clinical studies analyzing the gingival crevicular fluid (GCF) in periodontal health and disease. GCF represents the inflammatory exudate which bathes the gingival crevice or pocket, i.e., the space between the free gingiva and the tooth surfaces (25). GCF samples from periodontitis patients were shown to have complement-dependent hemolytic activity, suggesting that a functional complement system is present in this inflammatory exudate (26, 27). Activated complement fragments were shown to be highly abundant in the GCF from patients, but were undetectable or present in lower concentrations in GCF from healthy control individuals (28–32). Similarly, complement components and cleavage products were also readily detected in chronically inflamed gingiva but were undetected or at lower abundance in healthy tissue samples; the complement components detected in diseased gingiva (and also in GCF) were representative of the entire cascade (e.g., C1q, factor B, Bb, C3, C3a, C3b, C3c, C3d, C4, C5, C5a, C5b, C9) (26–37).

Importantly, periodontal therapy that resulted in decreased clinical indices of periodontal inflammation and tissue destruction also led to decreased C3 activation in the GCF (38). Conversely, and consistently, the progression of gingival inflammation during an experimental human gingivitis study was associated with elevated C3 cleavage in the GCF (32). Specifically, this study examined the cleavage of factor B, C3, and C4 in GCF collected during the experimental period and demonstrated, respectively, their conversion to Bb and C3c but not to C4c, thus implying selective activation of the alternative pathway (32). An immunohistochemical study showed that the complement regulator CD59 is expressed at lower levels in the gingiva of periodontitis patients as compared to healthy individuals, which might imply compromised protection of periodontitis-involved tissues against MAC-mediated autologous tissue damage (35).

More recent studies also support an association between complement and periodontitis. A case of aggressive periodontitis with severe angioedema localized to the gingiva was linked to dysregulated complement function, specifically deficiency of the C1-esterase inhibitor (C1INH) (39). A single nucleotide polymorphism affecting C5 (rs17611), which was previously linked to elevated C5 in serum and susceptibility to the complement-associated disease liver fibrosis (40), was shown to be more prevalent in patients with periodontitis than in healthy controls (41). In terms of its expression, C3 was shown to be among the top 5% genes that are most strongly downregulated following periodontal therapy (42). Another study has used integrative gene prioritization and databases from genome-wide association studies and microarray experiments, and identified C3 among the top 21 most promising candidate genes involved in periodontal disease (43). Interestingly, partial C4 gene deficiencies are significantly more frequent in periodontitis patients than in healthy individuals (44). This finding might suggest a protective function associated with C4, the activation of which occurs via the classical or the lectin pathways. However, it should be noted that C4a was recently shown to bind and activate protease-activated receptors (PAR) 1 and 4 (45) which are expressed by platelets and endothelial cells (46). Thus, C4-mediated effects may not necessarily involve downstream triggering of C3-dependent activities. Whether C4a might mediate complement crosstalk with the coagulation and/or the endothelial barrier system is currently uncertain as is the impact of such interactions on periodontitis.

The aforementioned clinical studies collectively indicate a role for complement activation in periodontal disease pathogenesis. However, the correlative nature of these human studies could not safely establish a causal relationship between complement and periodontitis and distinguish it from the alternative possibility that complement activation could simply be a marker of local periodontal inflammation. Causal evidence was derived from mechanistic animal model-based studies described below.

## Mechanistic Studies in Animal Models

Animals models can be engaged to determine causative links between potential mechanisms and disease pathogenesis (47), thereby not only promoting knowledge on pathogenesis but also identifying therapeutic targets and paving the way to human clinical trials. As the triggering of the complement cascade is intertwined with TLR activation (3), the two systems are discussed together in the studies presented here.

In response to microbial infection or tissue damage, complement and TLRs are swiftly activated, frequently by the same agonists. In this regard, bacterial lipopolysaccharide (LPS; a TLR4 agonist), fungal zymosan (TLR2/6 agonist) and bacterial CpG DNA (TLR9 agonist) not only induce TLR signaling but also can activate complement (48). In fact, complement and TLRs are not only co-activated in response to microbial infection and other types of insult, such as tissue injury, but they also engage in signaling crosstalk interactions in several myeloid cell types (monocytes, macrophages, neutrophils, and dendritic cells) (49–54) (Figure 1). In a pioneering study, different TLR agonists systemically given to mice lacking a major membrane-associated complement regulator, the decay-accelerating factor, induced significantly higher plasma levels of pro-inflammatory cytokines than wild-type controls (55). Similarly, mice systemically co-injected with TLR agonists (specifically TLR2, TLR4, and TLR9 ligands) and a potent complement activator (cobra venom factor) display remarkably high plasma levels of pro-inflammatory cytokines (55). In the complement-TLR crosstalk, the activated signaling pathways converge at mitogen-activated protein kinases (extracellular signal-regulated kinase-1 and-2 and c-Jun N-terminal kinase), which in turn activate key transcription factors, namely activator protein-1 (AP-1) and nuclear factor-κB (NF-κB) (55). Although this synergy has the potential to invigorate innate immunity against infection, it may also contribute to destructive inflammatory responses.

In line with these findings, the concomitant activation of C5aR1 and TLR2 in the mouse gingiva by local co-administration of specific ligands (C5a and Pam3Cys, respectively) resulted in the induction of significantly higher levels of IL-1β, IL-6, IL-17, and TNF than stimulation of either receptor alone (56). These data suggested that a synergy between complement and TLRs may be a major contributor to the induction of periodontal inflammation. In support of this notion, mice lacking C5aR1 are quite resistant against inflammatory bone loss regardless of the presence of TLR2 (57) and, in an analogous manner, mice lacking TLR2 are protected from inflammatory bone loss regardless of the presence of C5aR1 (58). TLR9-deficient mice are also protected against experimental periodontitis (59), which could be attributed, at least in part, to complement-TLR9 synergy (55). These studies utilized a murine model of periodontitis in which the disease is initiated by dysbiosis following oral gavage with the keystone pathogen *Porphyromonas gingivalis* (60, 61). Consistent with the importance of complement involvement in periodontal disease pathogenesis, C3-deficient mice were protected against periodontitis in three distinct models, ligature-induced periodontitis, *P. gingivalis*-induced periodontitis, and aging-associated periodontitis (62). The ligature model involves the placement of silk ligatures around molar teeth leading to massive accumulation of indigenous bacteria and induction of inflammation and alveolar bone loss in specific-pathogen-free (but not germ-free) animals (60, 63–65). In the aging-associated periodontitis model, periodontal inflammation and bone loss develops naturally as a function of old age when homeostatic mechanisms break down (66–68). Interestingly, C3-deficient mice also exhibited reduced periodontal bacterial load in *P. gingivalis*-induced periodontitis as compared to wild-type littermate controls (62). These data suggest that lack of complement activation does not lead to defective control of the periodontal microbiota and, moreover, are consistent with the concept that destructive inflammation is required to sustain a quantitatively and compositionally altered dysbiotic microbiota (18).

## Translational Preclinical Studies

The studies discussed earlier suggested that complement may be a promising target for the treatment of periodontitis. Indeed, in the oral gavage model of *P. gingivalis*-induced periodontitis, intra-gingival microinjection of wild-type mice with PMX-53, a C5aR1 antagonist (69), suppressed the induction of inflammatory cytokines (IL-1β, IL-6 and IL-17, and TNF) in the gingival tissue and inhibited alveolar bone loss (56). This protective effect occurred despite the presence of intact TLR2, in other words, inflammatory bone loss can be effectively inhibited by blocking only one of the two cross-talking receptors (56). PMX-53 was also tested in the ligature-induced periodontitis model where disease can be initiated independently of *P. gingivalis* (63). Although substantial inflammatory bone loss was induced after 5 days at the ligated areas of control-treated mice, mice locally microinjected (at the ligated sites) with PMX-53 exhibited significant protection against periodontal inflammation and bone loss (56). Rats given PMX-205 [another C5aR1 antagonist (70)] via the drinking water were also protected from ligature-induced bone loss (71), although with reduced efficacy perhaps due to the different route of drug administration and/or the use of a different animal species.

It is important to note that the same inflammatory mediators (e.g., TNF, IL-1β, prostaglandin E2) have been implicated in inflammatory periodontal bone loss across different species, such as mice, rats, dogs, non-human primates, and humans (72–77). Therefore, mice appear to be a useful model for human periodontitis especially for mechanistic studies, since mice currently represent the only available species with engineered knock-in or knock-out mutations for a whole panel of key immune response genes. However, promising results obtained in higher animals, such as non-human primates, increase the possibility that candidate drugs can be protective also in humans. In this regard, the periodontal tissue anatomy and immune system of non-human primates are similar to those of humans, and periodontitis in monkeys displays clinical, microbiological, and immuno-histological features that are highly similar to those of human periodontal disease (78–82). In fact, the use of non-human primates becomes necessary for testing drugs that lack specificity for the widely used rodent models and other small animals.

**Figure 2:**
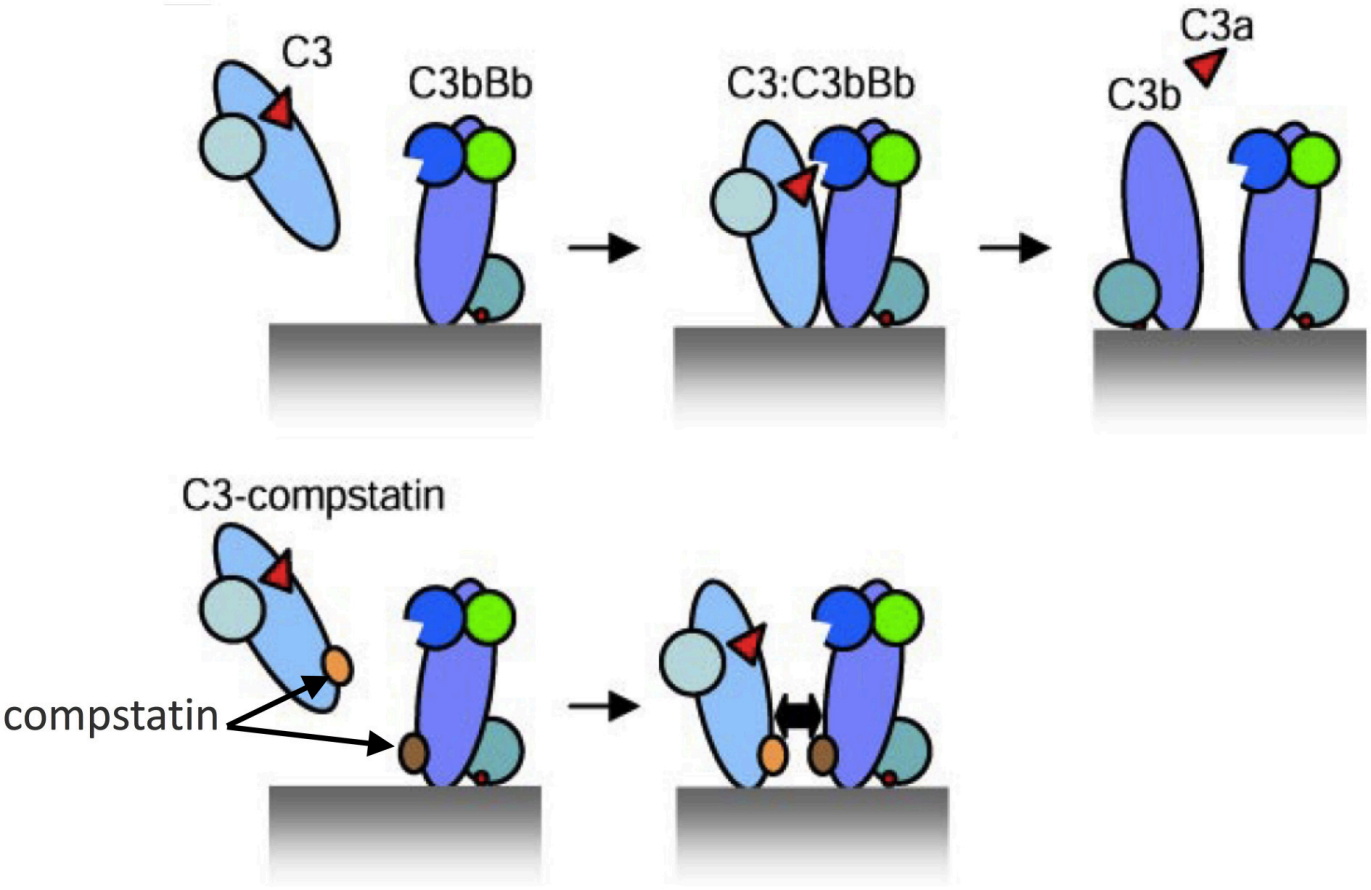
Model of C3 activation and its inhibition by compstatin. **(Top)** Depiction of key protein interactions resulting in the formation of C3 convertase on a target surface (e.g., a microbial cell surface). Native C3 binds to the convertase (C3bBb) and is cleaved into its active fragments, C3a and C3b. **(Bottom)** Compstatin acts by blocking protein-protein interaction. Specifically, compstatin binds both native C3 and C3b and sterically hinders the binding of native C3 by C3 convertases, hence preventing C3 cleavage into its active fragments. From ref. 87. Used by permission.

In this regard, compstatin and new generation analogs are small peptidic inhibitors that have an exquisite specificity for human and non-human primate C3 (83–85). Given the absence of available C3 inhibitors in mice, the appropriateness of C3 as a therapeutic target in periodontitis could only be tested in primates. Specifically, the third-generation compstatin analog Cp40 was tested in cynomolgus monkeys (*Macaca fascicularis*) (62). Cp40 has a subnanomolar affinity for C3 (K_D_ = 0.5 nM; 6,000-fold greater than that of the original compstatin) and a plasma human half-life (48 h) that exceeds expectations for most peptidic drugs. Mechanistically, the original compstatin and new generation analogs bind C3 and block its interaction with and cleavage by the C3 convertase into its active fragments, C3a and C3b (86) (Figure 2). In other words, the compstatin family of C3 inhibitors protect the C3 substrate rather than interfere with the C3 convertase. As a consequence, the compstatins prevent propagation and amplification of complement activation and generation of effector molecules regardless of the mechanism that initiated complement activation (83, 84).

Periodontitis in adult cynomolgus monkeys was induced by placing silk ligatures around posterior teeth on both halves of the mandible (lower jaw) for a period of 6 weeks. Local treatment (through intra-gingival injection) with Cp40 started 3 days following ligature placement. A split-mouth experimental design was applied, where one side was treated with Cp40 and the other with an inactive control peptide, thus each animal served as its own control. The disease was monitored clinically by analyzing clinical indices that assess periodontal inflammation and tissue destruction (87). Cp40 treatment resulted in significant decrease of gingival index and clinical attachment loss, which correlated with lower levels of proinflammatory and osteoclastogenic cytokines (e.g., TNF, IL-1β, IL-17, and RANKL) in the GCF, as well as with decreased numbers of osteoclasts in bone biopsy specimens (62). In contrast to RANKL, the GCF content of osteoprotegerin (OPG), a natural inhibitor of RANKL, was maintained at increased levels in Cp40-treated as compared to control sites. Therefore, Cp40 appeared to cause a favorable reversal of the RANKL/OPG ratio, which is a potential indicator of periodontitis (88). Consistent with these data, radiographic analysis showed that Cp40-treated sites had significantly less bone loss as compared to control-treated sites (62).

To determine the potential usefulness of Cp40 in a therapeutic setting, the drug was administered to adult cynomolgus monkeys with pre-existing, naturally-occurring chronic periodontitis (89). The animals were locally injected in the gingiva with Cp40 either once a week (group of 5 animals) or three times per week (group of 10 animals) for a 6 weeks treatment period followed by a 6 weeks follow-up period in the absence of Cp40 treatment. Clinical examinations and collections of GCF samples were conducted at baseline and throughout the study. In both groups, treatment with Cp40 led to significant decrease in clinical indices that assess periodontal inflammation (gingival index and bleeding on probing), tissue destruction (probing pocket depth and clinical attachment loss) or tooth mobility which is often linked to bone loss. The improvement of clinical disease as reflected by reduced clinical indices correlated with decreased levels of proinflammatory and osteoclastogenic mediators (e.g., IL-17 and RANKL) in the GCF and decreased osteoclast numbers in bone biopsies. The protective effects mediated by Cp40 endured, although with reduced effectiveness, for at least 6 weeks after the drug was discontinued. Cp40 could therefore reverse pre-existing chronic periodontal inflammation without additional treatments, such as scaling and root planing (SRP) (89). Proteomic analysis of GCF samples collected from that study showed involvement of both the alternative and classical pathways of complement activation in naturally occurring non-human primate periodontitis; however, the alternative pathway was the most enriched of all biological pathways identified by gene ontology analysis (90). These proteomic findings are consistent with early clinical reports indicating that the complement alternative pathway is predominantly activated in GCF samples from human periodontitis patients, although the classical pathway is also activated (28, 29, 38). Based on this consideration (and the likelihood that carbohydrate or glycoprotein components of periodontal bacteria may activate the lectin pathway) the concomitant inhibition of all three pathways (as can be done by Cp40) is likely to provide increased protection against periodontitis as compared to inhibition of individual pathways of complement activation. Another main target revealed by the proteomic fingerprinting of GCF samples from Cp40-treated NHPs was leukocyte degranulation. Neutrophils account for considerable tissue damage in human periodontitis, in great part through degranulation of tissue-degrading proteases and cytotoxic molecules (76, 91–94). In this regard, the ability of Cp40 to suppress exocytosis likely represents another host protective mechanism.

In a follow-up study in cynomolgus monkeys with naturally-occurring chronic periodontitis, it was shown that an effective therapeutic dose of locally administered Cp40 [100 μg/site; used in the study by Maekawa et al. (89)] does not cause local irritation and has long-lasting protective effects even when given as infrequently as once per 3 weeks (95). Therefore, taken together, clinical observations in humans and pre-clinical intervention studies in non-human primates suggest that complement is overactivated in periodontitis and that C3 inhibition by Cp40 is a promising host-modulatory therapy that warrants investigation as a potential treatment of human periodontitis.

**Figure 3:**
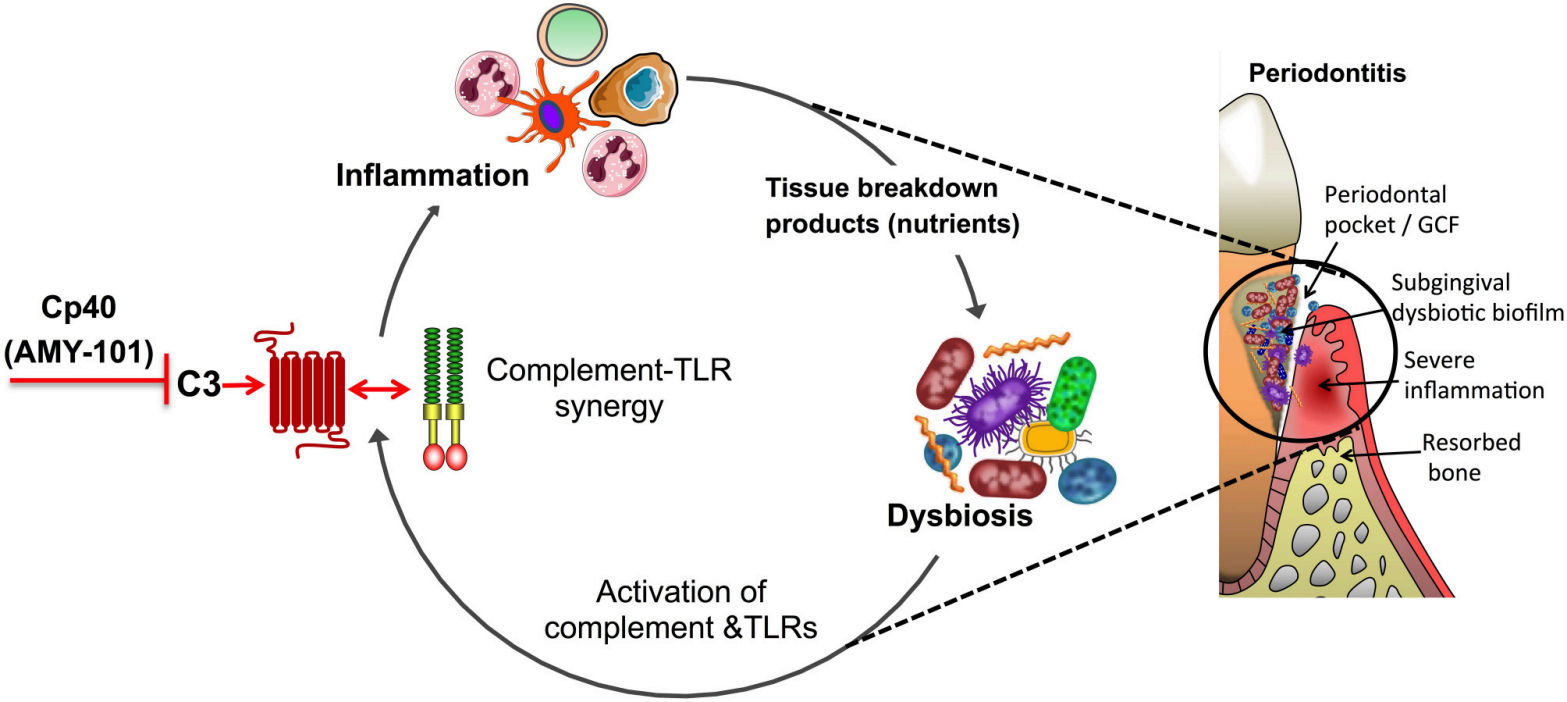
Complement-targeted inhibition blocks a vicious cycle linking destructive inflammation and dysbiosis in periodontitis. Periodontitis is driven by a reciprocally reinforced interplay between dysbiotic microbial communities and inflammation. Whereas, dysbiosis induces inflammation, the nutritionally favorable inflammatory environment is a major ecological factor that exacerbates dysbiosis. Studies in different preclinical models showed that complement inhibition abrogates complement-TLR crosstalk signaling and resulting inflammation, thereby breaking the disease-provoking vicious cycle regardless of the presence of intact TLR signaling. For instance, blocking the central complement component C3 through the use of the peptidic compound Cp40/AMY-101 has blocked experimental or naturally occurring periodontitis in non-human primates.

Given the potential for synergism between complement and TLRs, C3 inhibition in periodontitis can also inhibit inflammation that is activated by TLR signaling either in response to microbial TLR ligands (e.g., LPS, lipoproteins, and bacterial DNA) (96, 97) or in response to endogenous TLR ligands (e.g., biglycan, hyaluronan, or heparan sulfate fragments) that are released upon tissue injury and act as danger-associated molecular patterns (DAMPs) (98, 99). The latter suggests that complement inhibition may also suppress damaged tissue-induced inflammation, thereby blocking also the progression of periodontitis. Interestingly, several TLRs (TLR2, TLR3, TLR4, and TLR9), when activated by bacterial molecules or DAMPs released from stressed/damaged tissues, were shown to induce local expression of complement components (e.g., macrophage production of factor B and C3), thereby further promoting complement activity in an inflammatory environment (100–103). For instance, LPS induces production and release of factor B through a TLR4-TRIF pathway in macrophages (100). Moreover, TLR signaling suppresses the desensitization of G-protein-coupled receptors (GPCRs) by downregulating the expression of G-protein-coupled receptor kinases, required for inducing GPCR phosphorylation and internalization (104). This suggests a mechanism by which TLRs may potentially prolong the activation of GPCRs, such as C3aR and C5aR1. Furthermore, TLR-induced cytokines, such as IL-6, promote the expression of C3aR and C5aR (105). Therefore, TLRs regulate the expression of complement factors and both the expression and activation of complement receptors, which—as alluded to earlier—in turn can amplify TLR-dependent responses. This pro-inflammatory and potentially destructive feed-forward loop can be potentially disrupted by complement inhibition (Figure 3), a notion that may underlie the success of Cp40 treatment in the non-human primate preclinical model. Complement inhibition at the C3 level may also inhibit inflammasome-dependent inflammation since complement pathways (C3aR signaling and sublytic membrane attack complex) were shown to promote the activation of the NLRP3 inflammasome and IL-1β release (106, 107).

## Safety and Other Considerations for Clinical Use

Given the participation of the dysbiotic periodontal microbiota in the pathogenesis of periodontitis, the targeting of complement may not appear as an intuitive therapeutic option for this oral disease. In general, a potential concern regarding the therapeutic use of complement inhibitors is whether complement blockade may undermine the competency of host antimicrobial defenses and thus increase the risk of infection. Although this possibility may not be an issue in acute conditions that require transient complement inhibition [e.g., in hemodialysis (108)], it has to be carefully considered in conditions that will require long-term use of complement inhibitors. In this regard, individuals with primary C3 deficiencies have increased risk of certain infections (e.g., *Neisseria meningitidis* and *Streptococcus pneumoniae*) although this enhanced susceptibility appears to subside in adulthood, presumably owing to the development of compensatory defense mechanisms (109–111). Current experience from FDA-approved anti-complement drugs, such as eculizumab that blocks C5 activation, shows that immunization against encapsulated bacteria (such as meningococci) can largely diminish infectious risks. Therefore, vaccinations as well as prophylactic use of antibiotics may be included to enable safe use of complement inhibitors in chronic settings. Importantly, in cases of complement inhibition with small-molecule inhibitors, such as compstatin, the compound can more readily phased out (than an antibody for instance) if necessary, thus enabling swift recovery of complement-dependent antimicrobial functions. Importantly, the monitoring of non-human primates under prolonged (up to 3 months) systemic treatment with Cp40 revealed no significant differences in biochemical, hematological, or immunological parameters in their blood or tissues as compared to those of vehicle alone-treated controls, despite complete inhibition of C3 in the plasma. Intriguingly, moreover, wounds inflicted in the skin of the Cp40-treated animals did not show any signs of infection but rather exhibited a trend toward faster wound healing as compared with the vehicle-treated controls (112). This finding is consistent with earlier observations in mice in which C3 deficiency resulted in faster skin wound healing as compared to C3-sufficient control mice (113).

Although a chronic condition, periodontitis is a local inflammatory disease and thus can be treated via local complement inhibition, a much safer approach than systemic administration of the same inhibitor. Systemic exposure with complement inhibitors following local injection into the periodontal tissues should be negligible and thus not impair complement activity in circulation or other tissues. This notion can be exemplified by experience with Cp40. As C3 is the most abundant protein of the complement system in blood (1.0 to 1.5 mg/ml), small amounts of locally injected Cp40 that could “escape” from the periodontal tissue should be readily bound by excess C3 in blood, hence not reaching other tissues at active (inhibitory) concentrations. In the treatment regimen used in the above-described NHP study by Maekawa et al. (89), a total of 1.5 mg Cp40 was injected (15 sites at 100 μg/site). Even if the full local dose were to be given systemically, this would only result to an amount of 0.2–0.3 mg/kg bodyweight in non-human primates (0.02–0.03 mg/kg bodyweight in humans), whereas a systemic Cp40 dose of 1–2 mg/kg bodyweight is necessary to reliably attain target-exceeding drug levels in non-human primates (114).

Even at the local level, complement inhibition is unlikely to lead to defective control of the periodontitis-associated microbiota. As discussed above, C3-deficient mice have decreased periodontal bacterial load compared to C3-sufficient controls during experimental periodontitis (62). These data are consistent with the notion that inflammation is an ecological driver of dysbiosis in periodontitis (Figure 3). Indeed, destructive periodontal inflammation causes the generation of tissue breakdown products (such as degraded collagen peptides or heme-containing compounds) that are used as a nutrient source by a subset of bacterial species associated with dysbiosis; these are mainly proteolytic/asaccharolytic organisms with iron-acquisition mechanisms and/or can thrive by utilizing other inflammatory byproducts, such as nitrate for anaerobic respiration, thereby outcompeting health-associated bacteria and exacerbating dysbiosis (18, 115, 116). Therefore, complement inhibition by Cp40 may not simply inhibit inflammation but may additionally interfere with the outgrowth of the dysbiotic microbiota (Figure 3). Experimental support of the notion that anti-inflammatory approaches can have indirect anti-microbial effects has been obtained in mouse and rabbit models of periodontitis, where the control of inflammation not only protected against disease but also decreased the bacterial load and reversed dysbiosis (76, 77, 117–119). Conversely, and in line with the previous statement, the bacterial biomass of biofilms associated with human periodontitis increases with increasing clinical inflammation (120).

## Conclusions and Outlook

The studies discussed above suggest a clinical value of inhibiting all three main pathways of complement activation in periodontitis, which can be achieved by targeting the central component C3. C3 inhibition can directly inhibit inflammation and indirectly counteract dysbiosis. The safety and efficacy of Cp40 in non-human primate periodontitis (62, 89, 95) paves the way to clinical trials for the treatment of human periodontitis. To this end, aspects that need to be considered include questions regarding administration frequency, dosing, and selection of those patients who would most benefit from such a treatment. Even though Cp40 was successfully tested as a stand-alone treatment for both induced and naturally-occurring periodontitis in monkeys, the drug is more likely to be used as an adjunctive therapy to the management of human periodontal disease. Future clinical trials may investigate the combined potential of Cp40 and SRP to treat periodontal inflammation and suppress bone loss as compared to SRP alone. In very severe cases of periodontitis, combined Cp40 and SRP therapy could be compared to periodontal surgery, to determine whether the Cp40/SRP treatment can obviate the need for a surgical approach. It should be noted that a Cp40-based treatment (and host-modulation interventions in general) may not necessarily be applied in a therapeutic setting but also on a preventive basis (before the onset of periodontitis) to high-risk patients, such as cigarette smokers and diabetic patients (121–123).

The protective effects of Cp40 in non-human primate periodontitis are maintained for many weeks following drug withdrawal from treated monkeys (89, 95). This is an encouraging finding although the optimal frequency of Cp40 administration for long-term treatment of human periodontitis may need to be decided empirically. The unique pharmacokinetic properties of Cp40 described earlier are consistent with a “target-driven” model, where an initial rapid clearance of excess free peptide (i.e., not bound by C3) is followed by slow clearance of C3-bound peptide. The tight binding of Cp40 to C3 thus appears to delay its clearance and, indeed, the determined half-life values of different compstatin analogs correlate with their C3-binding affinities (85). Similarly, strong binding of Cp40 to abundant C3 in the inflamed periodontal tissue could contribute to delayed clearance of the drug from the tissue, thus contributing to sustained protection from periodontal inflammation. Moreover, the ability of Cp40 to arrest inflammation and presumably to reverse dysbiosis may reset the balance toward tissue homeostasis, which on its own (despite the absence of the drug) could resiliently inhibit or delay the recurrence of pathological processes.

Amyndas Pharmaceuticals is developing Cp40, a third-generation non-PEGylated compstatin analog, as the clinical candidate drug AMY-101. AMY-101 is developed for therapeutic interventions in C3 glomerulopathy (C3G), complications of ABO-incompatible kidney transplantation, paroxysmal nocturnal hemoglobinuria (PNH), and periodontal disease (124, 125). The PEGylated version of an earlier compstatin analog, POT-4/4(1MeW) (APL-2, Apellis Pharmaceuticals) is clinically developed for use in complement-mediated disorders including age-related macular degeneration and PNH. In a Phase II trial, prolonged APL-2 treatment was shown to be safe and reduced the growth rate of geographic atrophy associated with age-related macular degeneration. AMY-101 has obtained orphan drug designation for C3G and PNH from the U.S. Food and Drug Administration (FDA) and the European Medicines Agency (EMA) in 2016 and more recently, in 2017, successfully completed a phase I safety trial (125, 126). Targeted modifications of the *N*- and *C*-terminus of Cp40/AMY-101 have led to a series of fourth-generation compstatins with higher solubility, improved PK profiles thus broadening the spectrum of administration routes and likely reducing the dosing frequency of these peptidic drugs in chronic regimens (127). Overall, more than 20 candidate complement-targeted drugs that inhibit distinct points of the cascade are currently being tested in clinical trials for a variety of inflammatory and degenerative diseases (125). The documented safety of Cp40/AMY-101 and its protective effects in highly relevant preclinical models of periodontitis merits investigation in future clinical trials for the treatment of human periodontitis.

## Author Contributions

GH and JL: conceptualization; GH: original draft. All authors listed made a substantial intellectual contribution to the manuscript and edited and approved it for publication.

### Conflict of Interest Statement

JL is the founder of Amyndas Pharmaceuticals, which is developing complement inhibitors (including third-generation compstatin analogs, such as AMY-101). JL and GH are inventors of patents or patent applications that describe the use of complement inhibitors for therapeutic purposes, some of which are developed by Amyndas Pharmaceuticals. JL is also the inventor of the compstatin technology licensed to Apellis Pharmaceuticals [i.e., 4(1MeW)7W/POT-4/APL-1 and PEGylated derivatives]. The remaining authors declare that the research was conducted in the absence of any commercial or financial relationships that could be construed as a potential conflict of interest.
